# Two-dose aflibercept 8 mg versus three-dose aflibercept 2 mg loading for treatment-naïve neovascular age-related macular degeneration

**DOI:** 10.1371/journal.pone.0349947

**Published:** 2026-09-11

**Authors:** Mai Shirakami, Manabu Miyata, Masayuki Hata, Sotaro Ooto, Hiroshi Tamura, Ai Otsuka, Lin Tsukui, Naoko Ueda-Arakawa, Masahiro Miyake, Ayako Takahashi, Shin Kadomoto, Yuki Muraoka, Akitaka Tsujikawa

**Affiliations:** Department of Ophthalmology and Visual Sciences, Kyoto University Graduate School of Medicine, Kyoto City, Kyoto Prefecture, Japan; Shinshu University School of Medicine, JAPAN

## Abstract

This retrospective study compared the short-term efficacy of a two-dose intravitreal aflibercept (IVA) 8-mg loading regimen (2024–2025) with a conventional three-dose IVA 2-mg regimen (2013–2014) for treatment-naïve neovascular age-related macular degeneration (nAMD). Using 1:1 propensity score matching for age, sex, nAMD subtype, and baseline visual acuity, we evaluated 31 eyes per group. The primary endpoint was the proportion of eyes achieving a fluid-free center subfield one month after completing the respective loading phase (at 2 and 3 months from baseline, respectively). The fluid-free proportion was numerically higher in the 8-mg group (90.3%; 95% confidence interval [CI], 74.2–98.0%) than in the 2-mg group (80.6%; 95% CI, 62.5–92.5%); however, the difference was not statistically significant (*P* = 0.28). Both regimens yielded significant reductions in central retinal thickness, central choroidal thickness, and pigment epithelial detachment height, with no significant between-group differences. Within the 8-mg group, baseline intraretinal fluid strongly correlated with one-month visual acuity (r = 0.61, *P* < 0.001). These exploratory findings suggest that a reduced two-dose aflibercept 8-mg loading regimen provided short-term anatomical outcomes that did not differ significantly from the standard three-dose 2-mg regimen. This shortened loading phase potentially reduces the initial treatment burden. Further prospective studies should validate these preliminary outcomes.

## Introduction

Neovascular age-related macular degeneration (nAMD) is a major cause of irreversible central vision loss globally, and its prevalence is increasing due to an aging population [1,2]. Anti-vascular endothelial growth factor (VEGF) therapy is effective and safe for nAMD [1,3]. Among various anti-VEGF agents, aflibercept 2 mg has been widely used worldwide for over a decade, and its share reached approximately 80% between 2014 and 2020 in Japan [4]. Furthermore, studies have reported the actual long-term effectiveness and safety of intravitreal aflibercept (IVA) 2 mg; [5] however, the patient burden of frequent injections and subsequent cost remains a critical challenge, although the burden is lower than that of ranibizumab [6]. The burden is likely to induce drop-out [7].

To reduce the patient burden associated with frequent intravitreal anti-VEGF injections, aflibercept 8 mg was developed and is commercially available. The PULSAR trial (n = 1011 globally) demonstrated that aflibercept 8 mg with extended dosing intervals (every 12 or 16 weeks [q12w or q16w]) is non-inferior to aflibercept 2 mg with an 8-week (q8w) dosing interval in terms of efficacy and safety [8]. Although the standard regimen of all available agents as a loading therapy is three consecutive monthly doses, the increased potency of the aflibercept 8 mg may allow for a reduction. Direct comparative data supporting a reduced IVA 8 mg two-dose loading regimen against the conventional three-dose IVA 2 mg regimen are lacking.

Since retinal fluid affects the visual outcome in nAMD, [9] achieving fluid-free status is important. Large randomized controlled trials have assessed the proportion of eyes achieving a fluid-free status [8,10,11]. Here, this study aims to compare the short-term outcomes of a reduced two-dose loading regimen of IVA 8 mg with those of the conventional three-dose loading regimen of IVA 2 mg at 1 month after completing their respective loading phases, focusing on the fluid-free proportion in the center subfield as the primary endpoint.

## Methods

The ethics committee of the Kyoto University Graduate School of Medicine (Kyoto, Japan) approved this retrospective, observational study (approval number, R0532). All study protocols adhered to the tenets of the Declaration of Helsinki, and written informed consent was obtained from all participants. We accessed the data for research purposes from 12 June 2025 to 17 October 2025. We had no access to information that could identify individual participants after data collection.

### Participants

This study enrolled consecutive patients with the inclusion criteria as follows: (1) diagnosis of treatment-naïve nAMD by retinal specialists after institutional expert meetings; (2) age ≥ 50 years at the time of initial treatment; (3) presence of exudates involving the fovea on optical coherence tomography (OCT), defined as the presence of intraretinal fluid (IRF) or subretinal fluid (SRF), including mild subretinal hemorrhage (SRH) and subretinal fibrin; and (4) receiving two monthly loading doses of IVA 8 mg (Eylea 8 mg; Bayer, Leverkusen, Germany) initiated between June 2024 and May 2025 or three monthly loading doses of IVA 2 mg (Eylea 2 mg; Bayer) initiated between February 2013 and February 2014 at Kyoto University Hospital. During this 2024–2025 period, the two-dose loading regimen of IVA 8 mg was adopted as a uniform institutional protocol for all eligible patients, rather than being selected based on individual physician discretion. This strategy was prospectively implemented based on the presumed higher potency of the 8-mg formulation, aiming to reduce the initial treatment burden while achieving rapid fluid resolution. The exclusion criteria were as follows: (1) high myopia, defined as an axial length ≥ 26.5 mm; (2) other retinal diseases that could affect best-corrected visual acuity (BCVA), including epiretinal membrane (ERM) of Govetto’s stage 2–4, [12] retinal vein/artery occlusion, or diabetic retinopathy; (3) inability to perform necessary examinations or poor-quality imaging (e.g., due to dementia or severe vitreous hemorrhage); (4) undergoing cataract surgery during the studied period (baseline to primary endpoint visit); (5) non-Japanese ethnicity; or (6) cystoid macular degeneration. When both eyes of a patient met the criteria, we included the eye that received the initial treatment earlier in the analysis.

### Propensity score matching and group assignment

To minimize selection bias and confounding as much as possible, we used propensity score (PS) matching. The model included the clinically relevant baseline covariates of age, sex, nAMD subtype (typical nAMD, polypoidal choroidal vasculopathy, or retinal angiomatous proliferation), and baseline BCVA. The PSs were calculated using binary logistic regression analysis, and we matched eyes receiving IVA 8 mg (8-mg group) 1:1 to eyes receiving IVA 2 mg (2-mg group) using nearest-neighbor matching.

#### Data collection.

Before treatment, we essentially performed a comprehensive ophthalmological examination, including BCVA assessment using a Landolt chart (with values converted to Snellen equivalents and the logarithm of the minimum angle of resolution [logMAR] for statistical analysis), refractive error measurement, axial length measurement using partial coherence interferometry (IOLMaster; Carl Zeiss Meditec, Dublin, USA), OCT imaging (Spectralis; Heidelberg Engineering, Heidelberg, Germany) covering a 30° field centered on the fovea, using cross-scans (automatic real-time tracking [ART], 100 frames) and horizontal raster scans consisting of 13 lines within the central vertical 10° (ART, 50 frames), color fundus photography (TRC-NW8F; Topcon, Tokyo, Japan), and fluorescein and indocyanine green angiography (Spectralis HRA; Heidelberg Engineering). Retinal specialists diagnosed nAMD and its subtypes (typical nAMD, PCV, and RAP) and decided on the treatment. At 1 month after loading therapy, we performed follow-up examinations.

### OCT analysis

A single investigator (MS) performed all OCT analyses using B-scan images through the fovea with the built-in software. The investigator measured central retinal thickness (CRT) as the distances between the inner border of the internal limiting membrane and the inner border of the retinal pigment epithelium (RPE) at the fovea, central choroidal thickness (CCT) as the distance between the outer surface of Bruch’s membrane and the chorioscleral interface at the subfovea, and subfoveal pigment epithelial detachment (PED) height as the distance between the outer surface of the RPE and the inner surface of Bruch’s membrane at the subfovea at baseline and 1 month after the loading phase. She investigated the presence of IRF and SRF, including SRH and subfoveal fibrin at baseline in the center subfield, defined as a circle with a 1 mm diameter centered on the fovea. Regarding subretinal hyperreflective material (SHRM), defined as hyperreflective material located external to the retina and internal to the RPE, [13] she referred to color fundus photographs to distinguish its components. Consistent with the components described previously,^13^ SHRM corresponding to reddish lesions was classified as SRH, while SHRM corresponding to yellowish-white lesions was defined as subretinal fibrin. Furthermore, she assessed for a fluid-free (dry) center subfield, defined as the absence of both IRF and SRF in the center subfield, as previously reported, [8] on OCT images at 1 month after the loading therapy. In this assessment, residual exudative activity outside the central 1-mm area or isolated sub-RPE fluid (i.e., PED without overlying SRF or IRF) did not preclude classification as fluid-free for the primary endpoint.

#### Primary and secondary endpoints.

We defined the evaluation point as 1 month after the final loading injection for both groups (i.e., 2 months after baseline for the 8-mg group and 3 months after baseline for the 2-mg group). Although comparing groups at an identical chronological time point from baseline is an alternative, we prioritized a fair comparison of the anatomical response by unifying the interval after the final loading dose to 1 month for both regimens. The primary endpoint was the proportion of eyes achieving a fluid-free center subfield at 1 month after the loading therapy. Secondary endpoints included changes in the logMAR BCVA, CRT, CCT, and subfoveal PED height from baseline to 1 month after the loading therapy. We calculated these changes by subtracting the value at 1 month from the baseline value. Furthermore, we evaluated the incidence of any adverse events during this period as a secondary safety endpoint.

### Statistical analysis

We primarily performed statistical analyses using SPSS (version 27.0; IBM, Armonk, NY, USA). We presented data as the mean ± standard deviation, where applicable. We compared the parameters between the two groups using the Mann-Whitney U test or chi-square test, as appropriate, and compared parameters between baseline and 1 month after the loading therapy using the Wilcoxon signed rank test. We calculated the 95% confidence intervals (CIs) for the proportions of eyes achieving a fluid-free center subfield using the exact binomial (Clopper-Pearson) method. We used R software (version 4.5.1; R Foundation for Statistical Computing, Vienna, Austria) solely to compute the 95% CI for the between-group difference using Newcombe's method, as this procedure is not natively supported by SPSS. As a subanalysis to identify prognostic factors in the 8-mg group, we assessed correlations between logMAR BCVA at 1 month and baseline parameters using Spearman’s rank correlation coefficient; furthermore, we compared baseline characteristics between fluid-free eyes and fluid-persistent eyes in the 8-mg group using the Mann-Whitney U test or chi-square test, as appropriate. Covariate balance between the two groups before and after matching was evaluated using absolute standardized mean differences (SMDs). We considered *P* values < 0.05 statistically significant.

## Results

From the consecutive patients, we identified 31 eligible eyes of 31 patients in the 8-mg group after excluding 4 eyes (axial length ≥ 26.5 mm, n = 1; non-Japanese ethnicity, n = 2; cystoid macular degeneration, n = 1) and 82 eligible eyes of 82 patients in the 2-mg group after excluding 4 eyes (cataract surgery during the study period, n = 2; non-Japanese ethnicity, n = 1; cystoid macular degeneration, n = 1). Following treatment initiation, no patients in the 8-mg cohort dropped out or required exclusion before the primary endpoint evaluation. Using 1:1 propensity score matching, we matched all 31 eyes in the 8-mg group with 31 eyes selected from the 82 eligible eyes in the 2-mg group. The C-statistic for the PS model was 0.64. Before matching, the age was significantly higher in the 8-mg cohort than in the 2-mg cohort (*P* = 0.03), with a substantial imbalance (SMD, 0.48) (S1 Table). However, after matching, the age was perfectly balanced (SMD, 0.004), and the baseline characteristics of the 8-mg and 2-mg groups showed no significant differences (Table 1). Specifically, demographic parameters were comparable, including age (78.8 ± 7.6 vs. 78.8 ± 7.4, *P* = 0.93), sex distribution (18 men/13 women vs. 19 men/12 women, *P* = 0.80), and nAMD subtype (*P* = 0.56). Baseline functional and morphological parameters were also comparable, including logMAR BCVA (0.44 ± 0.41 [Snellen equivalent, 20/55] vs. 0.39 ± 0.32 [20/50], *P* = 0.96), CRT (361.8 ± 147.0 μm vs. 350.4 ± 179.5 μm, *P* = 0.56), CCT (194.5 ± 50.2 μm vs. 203.7 ± 79.8 μm, *P* = 0.68), and subfoveal PED height (90.9 ± 173.1 μm vs. 87.5 ± 180.0 μm, *P* = 0.76). Thus, the baseline parameters were well-matched.

**Table 1:** Comparison of the studied parameters between the 8-mg and 2-mg groups.

		8-mg group	2-mg group	*P* value	SMD
Eyes, n		31	31		
Age at baseline, years		78.8 ± 7.6	78.8 ± 7.4	0.93	0.004
Sex, men/women, n		18/13	19/12	0.80^†^	0.067
Subtype, typical nAMD/PCV/RAP, n		14/14/3	18/10/3	0.56^†^	0.277/0.252/0
Spherical equivalence at baseline, diopters		+0.42 ± 2.47	+0.44 ± 1.69	0.83	0.009
Axial length, mm		23.95 ± 1.11^a^	23.36 ± 1.05^b^	0.08	0.543
LogMAR BCVA	Baseline	0.44 ± 0.41 (Snellen, 20/55)	0.39 ± 0.32 (Snellen, 20/50)	0.96	0.114
	1 month after loading therapy	0.41 ± 0.37 (Snellen, 20/51)	0.37 ± 0.50 (Snellen, 20/47)	0.35	0.085
	Change (baseline−post-loading)	0.03 ± 0.24	0.02 ± 0.36	0.22	0.012
CRT, μm	Baseline	361.8 ± 147.0	350.4 ± 179.5	0.56	0.070
	1 month after loading therapy	176.2 ± 73.0	187.6 ± 110.4	0.65	0.122
	Change (baseline−post-loading)	185.6 ± 137.7	162.8 ± 213.6	0.55	0.127
CCT, μm	Baseline	194.5 ± 50.2	203.7 ± 79.8	0.68	0.137
	1 month after loading therapy	174.6 ± 52.5	171.0 ± 67.0	0.98	0.059
	Change (baseline−post-loading)	19.9 ± 31.1	32.6 ± 55.1	0.56	0.284
Subfoveal PED height, μm	Baseline	90.9 ± 173.1	87.5 ± 180.0	0.76	0.019
	1 month after loading therapy	34.1 ± 74.3	15.7 ± 24.0	0.44	0.335
	Change (baseline−post-loading)	56.8 ± 135.1	71.8 ± 178.1	0.67	0.095

Data are presented as means ± standard deviations where applicable.

nAMD = neovascular age-related macular degeneration; PCV = polypoidal choroidal vasculopathy; RAP = retinal angiomatous proliferation; LogMAR BCVA = logarithm of the minimum angle of resolution best-corrected visual acuity; CRT = central retinal thickness; CCT = central choroidal thickness; PED = pigment epithelial detachment; SMD = standardized mean difference.

The data for ^a^ and ^b^ were missing in 2 and 2 eyes, respectively.

^†^, Chi-square test; for the others, the Mann–Whitney U test.

### Primary and secondary endpoints

**Fig 1:**
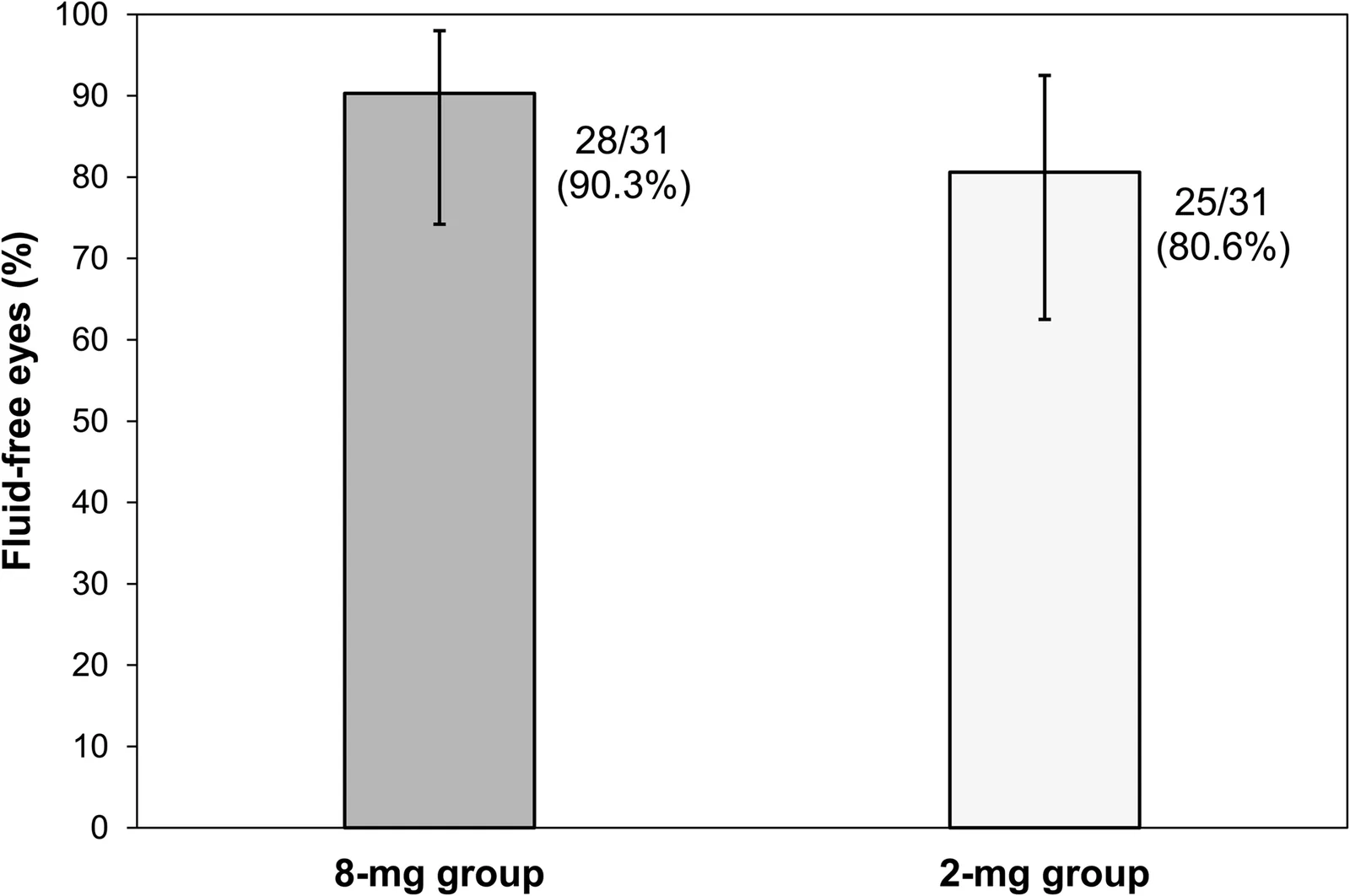
Proportion of eyes achieving fluid-free center subfield at 1 month after loading therapy. The proportion of eyes achieving a fluid-free center subfield was higher in the 8-mg group (28/31 eyes; 90.3%) than in the 2-mg group (25/31 eyes; 80.6%); however, this difference did not reach statistical significance (*P* = 0.28). Error bars represent 95% confidence intervals calculated using the exact binomial (Clopper-Pearson) method.

The proportion of eyes achieving a fluid-free center subfield (primary endpoint) was higher in the 8-mg group (28 of 31 eyes; 90.3%, 95% confidence interval [CI], 74.2–98.0%) than in the 2-mg group (25 of 31 eyes; 80.6%, 95% CI, 62.5–92.5%); the between-group difference was 9.7% (95% CI, −8.8–28.5%), which did not reach statistical significance (*P* = 0.28) (Fig 1). Regarding the secondary endpoints, the changes from baseline to 1 month after the loading phase did not differ significantly between the two groups (Table 1). The change in logMAR BCVA was 0.03 ± 0.24 in the 8-mg group and 0.02 ± 0.36 in the 2-mg group (*P* = 0.22). BCVA did not change significantly in either the 8-mg or 2-mg group (*P* = 0.51 and 0.057, respectively). Similarly, morphological changes, including CRT (185.6 ± 137.7 μm vs. 162.8 ± 213.6 μm, *P* = 0.55), CCT (19.9 ± 31.1 μm vs. 32.6 ± 55.1 μm, *P* = 0.56), and subfoveal PED height (56.8 ± 135.1 μm vs. 71.8 ± 178.1 μm, *P* = 0.67), did not differ significantly between the groups (Table 1). However, these morphological parameters showed significant reductions from baseline in both the 8-mg group (CRT, *P* < 0.001; CCT, *P* = 0.002; PED height, *P* = 0.03) and the 2-mg group (CRT, *P* < 0.001; CCT, *P* = 0.003; PED height, *P* = 0.004). Concerning safety, no agent-associated adverse events occurred in either group during the study period.

### Subanalysis in the 8-mg group

**Fig 2:**
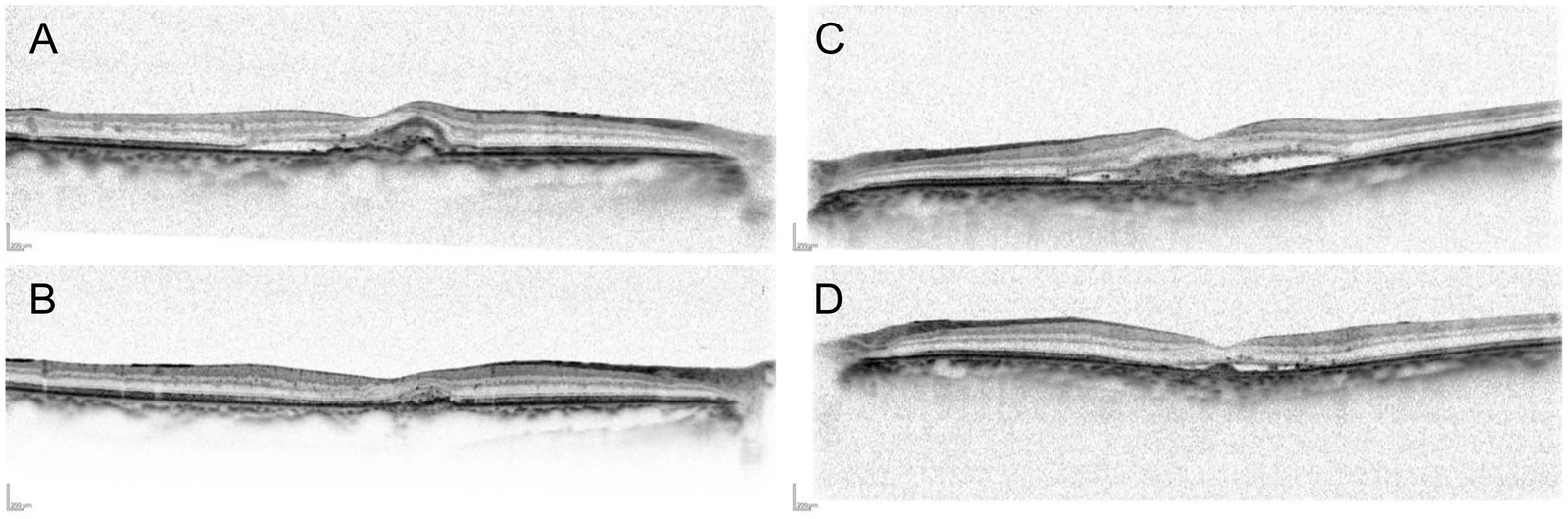
Representative optical coherence tomography images of eyes with subretinal fibrin at baseline and classified as fluid-persistent 1 month after two loading doses in the 8-mg group. (A, B) Horizontal B-scan images of the right eye in a woman in her late 70s. At baseline (A), the image shows subretinal fibrin and subretinal fluid (SRF). One month after two loading doses of aflibercept 8 mg (B), the SRF resolved, but a small amount of fibrin persisted (classified as fluid-persistent). (C, D) Horizontal B-scan images of the left eye in a man in his late 80s. At baseline (C), the image shows subretinal fibrin and SRF. One month after two loading doses of aflibercept 8 mg (D), the subretinal fibrin resolved, but a small amount of SRF persisted (classified as fluid-persistent).

In the 8-mg group, the logMAR BCVA at 1 month significantly correlated with baseline logMAR BCVA (r = 0.87, *P* < 0.001), baseline CRT (r = 0.37, *P* = 0.04), subfoveal PED height (r = 0.39, *P* = 0.03), and the presence of IRF (r = 0.61, *P* < 0.001) (Table 2). Furthermore, fluid-persistent eyes (n = 3) tended to have a higher prevalence of subretinal fibrin (Fig 2) compared to fluid-free eyes (n = 28) (67% [2/3] vs. 21% [6/28], *P* = 0.09) (Table 3). Other baseline parameters did not differ significantly between the two subgroups.

**Table 2:** Correlations between the 1-month logMAR BCVA after loading dose and baseline parameters in the 8-mg group.

Baseline parameters	r	*P* value
Age	0.25	0.17
Sex (1, men; 2, women)	−0.24	0.19
Subtype (1, typical nAMD; 2, PCV; 3, RAP)	−0.12	0.51
Spherical equivalence	0.29	0.12
Axial length^a^	−0.14	0.49
LogMAR BCVA	0.87	< 0.001*
CRT	0.37	0.04*
CCT	−0.05	0.80
Subfoveal PED height	0.39	0.03*
SRF (0, − ; 1, +)	0.02	0.91
IRF (0, − ; 1, +)	0.61	< 0.001*
SRH (0, − ; 1, +)	0.08	0.68
Subretinal fibrin (0, − ; 1, +)	0.02	0.93

LogMAR BCVA = logarithm of the minimum angle of resolution best-corrected visual acuity; nAMD = neovascular age-related macular degeneration; PCV = polypoidal choroidal vasculopathy; RAP = retinal angiomatous proliferation; CRT = central retinal thickness; CCT = central choroidal thickness; PED = pigment epithelial detachment; SRF = subretinal fluid; IRF = intraretinal fluid; SRH = subretinal hemorrhage.

The data for ^a^ were missing in 2 eyes.

* Statistically significant (*P* < 0.05).

**Table 3:** Comparison of baseline parameters between fluid-free eyes and fluid-persistent eyes in the 8-mg group.

	Fluid-free	Fluid-persistent	*P* value
Eyes, n	28	3	
Age, years	78.7 ± 7.8	80.3 ± 5.8	0.64
Sex, men/women, n	16/12	2/1	0.75^†^
Subtype, typical nAMD/PCV/RAP, n	13/12/3	1/2/0	0.68^†^
Spherical equivalence, diopters	+0.56 ± 2.23	−0.88 ± 4.67	0.59
Axial length, mm	23.87 ± 1.09^a^	24.68 ± 1.24	0.25
LogMAR BCVA	0.41 ± 0.41 (Snellen, 20/51)	0.64 ± 0.39 (Snellen, 20/87)	0.29
CRT, μm	352.9 ± 122.3	445.7 ± 332.2	0.93
CCT, μm	190.3 ± 47.1	234.3 ± 72.6	0.32
Subfoveal PED height, μm	98.4 ± 180.5	21.0 ± 36.4	0.55
SRF, absent/present, n	8/20	0/3	0.28^†^
IRF, absent/present, n	19/9	2/1	0.97^†^
SRH, absent/present, n	23/5	2/1	0.52^†^
Subfoveal fibrin, absent/present, n	22/6	1/2	0.09^†^

nAMD = neovascular age-related macular degeneration; PCV = polypoidal choroidal vasculopathy; RAP = retinal angiomatous proliferation; logMAR BCVA = logarithm of the minimum angle of resolution best-corrected visual acuity; CRT = central retinal thickness; CCT = central choroidal thickness; PED = pigment epithelial detachment; SRF = subretinal fluid; IRF = intraretinal fluid; SRH = subretinal hemorrhage.

The data for ^a^ were missing in 2 eyes.

^†^, Chi-square test; for the others, the Mann–Whitney U test.

## Discussion

This PS-matched study found that a two-dose loading regimen of IVA 8 mg provided short-term anatomical and functional outcomes that did not differ significantly from the conventional three-dose loading regimen of IVA 2 mg in treatment-naïve eyes with nAMD. Specifically, the 8-mg group achieved a high proportion of fluid-free center subfield (90.3%), which was numerically higher than that of the 2-mg group (80.6%), although the difference did not reach statistical significance. Furthermore, both groups exhibited significant improvements in morphological parameters, including CRT, CCT, and subfoveal PED height, without significant differences between the two groups. These exploratory findings suggest that the enhanced potency of the 8 mg formulation may allow a reduced initial injection frequency during the loading phase while achieving short-term fluid resolution that did not differ significantly from the conventional regimen, potentially offering a more efficient induction strategy for treatment-naïve nAMD.

Regarding the primary endpoint, the 8-mg group achieved a numerically higher proportion of fluid-free center subfield after only two loading doses than the 2-mg group after three loading doses (90.3% vs. 80.6%, *P* = 0.28). Although evaluating only the center subfield does not fully capture overall disease activity, this trend aligns with the pivotal PULSAR trial, where the proportion in the IVA 8 mg group was significantly higher than in the IVA 2 mg group at 2 months after the three loading doses (63% vs. 52%, *P* = 0.002), a trend consistent with our findings [8]. However, at week 96, the proportion became comparable between IVA 8 mg and 2 mg (66.5% vs. 66.6%), [14] which is likely attributable to a ceiling effect. Taken together, these findings suggest that a primary advantage of IVA 8 mg is the rapid resolution of fluid with fewer injections compared with IVA 2 mg. The ALTAIR trial showed that 2-year BCVA in eyes without fluid at 2 months after three loading doses of IVA 2 mg was better than that in eyes with persistent fluid [9]. Consequently, in addition to these benefits, the two-dose 8 mg regimen could offer the advantage of enabling earlier assessment of anatomical response.

The proportion of fluid-free center subfield after the loading phase in our 8-mg group was numerically higher than in the PULSAR trial (90% vs. 63%) [8]. A potential reason for the higher proportion in this study may be Japanese ethnicity. A multicenter international study showed that the subtype proportion of nAMD differed substantially between Japanese and French patients using the same diagnosis criteria (PCV, 48% vs. 9%) [15]. Furthermore, a meta-analysis showed that the reduction in CRT at 3 months after anti-VEGF therapy was significantly greater in the PCV group compared to the non-PCV group (*P* = 0.03) [16]. Moreover, another study showed that Japanese patients with PCV achieved superior 6-year outcomes compared with non-Japanese patients with PCV [17]. While the PULSAR trial included various ethnicities and was predominantly White (approximately 75%), this study included only Japanese patients. Consequently, the higher PCV proportion in our cohort likely contributed to the more favorable anatomical outcomes. Another potential factor is the difference in inclusion criteria. Although the PULSAR trial restricted BCVA to 20/32–20/320, this study did not set strict BCVA criteria, except for excluding cystoid macular degeneration (advanced stage). Therefore, disease severity might differ; nevertheless, the mean baseline logMAR was comparable between the two studies (PULSAR, approximately 0.5; this study, 0.44).

Morphologically, the 8-mg group exhibited significant reductions in CRT, CCT, and subfoveal PED height after two loading doses, which were similar to those observed in the 2-mg group after three loading doses. These findings suggest that the high-dose formulation has sufficient potency to rapid improve macular morphology with fewer injections. Notably, although the injection frequency was reduced, the total drug load administered during the loading phase was substantially higher in the 8-mg group (16 mg; 8 mg × 2) compared with the 2-mg group (6 mg; 2 mg × 3). This higher cumulative dose likely compensates for the reduced frequency, contributing to the rapid anatomical improvement. A previous analysis of the CATT and IVAN trials showed that greater CRT fluctuation (SD calculated from 4 or more time-point measurements) during anti-VEGF treatment for nAMD is associated with worse BCVA and the development of fibrosis and macular atrophy, suggesting that maintaining a stable fluid-free macula is the treatment target [18]. Therefore, the potent initial fluid resolution achieved with aflibercept 8 mg may help achieve such structural stability.

Apart from baseline BCVA (r = 0.87, *P* < 0.001), which is expected, the subanalysis in the 8-mg group identified baseline IRF (r = 0.61, *P* < 0.001) as a potential factor associated with 1-month BCVA. This suggests that the presence of IRF reflects preexisting neurosensory retinal damage that limits BCVA recovery, as previously reported [19,20]. However, because the small sample size precluded multivariable adjustment, this correlation may largely reflect baseline BCVA and overall baseline disease severity. Furthermore, fluid-persistent eyes tended to have a higher prevalence of subretinal fibrin compared with fluid-free eyes (67% vs. 21%, *P* = 0.09). Although the extremely small number of fluid-persistent eyes (n = 3) makes this finding preliminary, subretinal fibrin is a major component of subretinal hyperreflective material (SHRM), a known morphological biomarker for developing subretinal fibrosis [13]. The presence of fibrin may act as a physical barrier to fluid absorption or indicate more severe inflammation, potentially limiting the efficacy of the initial loading doses even with the high-dose 8 mg formulation. Such cases warrant careful monitoring for attenuated therapeutic responses.

This study has several limitations. First, its retrospective nature may introduce selection bias. Although propensity score matching balanced demographics and baseline visual acuity, the model did not include anatomical variables to prevent overfitting due to sample size constraints, leaving potential residual confounding. Nevertheless, good covariate balance was confirmed after matching for key unmatched anatomical parameters such as CRT and PED height, as demonstrated by the SMDs. Second, the sample size was relatively small. Although the 8-mg group achieved a numerically higher fluid-free rate than the 2-mg group (90.3% vs. 80.6%), the study was underpowered to establish statistical significance. Therefore, this non-significant result should not be interpreted as clinical equivalence. Additionally, despite no short-term agent-associated adverse events, the small cohort size means that the overall safety profile—particularly regarding uncommon but important adverse events—cannot be meaningfully assessed. Third, evaluating the primary endpoint one month after completing the respective loading phases creates a chronological discrepancy from baseline (2 months for the 8-mg group vs. 3 months for the 2-mg group). Because routine injections are often outsourced to affiliated local clinics, 2-month morphological data for the 2-mg group were largely unavailable. Thus, this study evaluates only the post-loading status and cannot prove earlier disease control. Furthermore, this short follow-up period precludes conclusions regarding the long-term stability of the fluid-free status. Fourth, we excluded eyes with massive SMH, because we routinely perform displacement by vitrectomy with subretinal tissue plasminogen activator, [21,22] given that a multicenter survey showed the superiority of displacement for massive submacular hemorrhage with BCVA worse than 20/40 compared with anti-VEGF monotherapy [23]. Therefore, our findings better reflect real-world clinical use of aflibercept. Fifth, the more than 10-year gap between the 2-mg cohort (2013–2014) and the 8-mg cohort (2024–2025) may introduce a chronological bias. We selected the historical cohort primarily because adherence to the standard three-dose loading protocol for consecutive patients with treatment-naïve nAMD was strictest during that period. Recognizing the necessity of robust cross-era comparisons, our institution intentionally maintained the identical spectral-domain OCT device (Spectralis) and standardized scanning protocols over the decade to minimize technological bias. Nevertheless, propensity score matching cannot fully correct for this period effect. Unmeasured temporal changes over this decade, including evolving diagnostic thresholds, OCT interpretation, clinical decision-making, patient referral patterns, and treatment philosophy for nAMD, may have influenced the outcomes.

In conclusion, these exploratory findings suggest that a reduced two-dose aflibercept 8-mg loading regimen provided short-term anatomical outcomes that did not differ significantly from the standard three-dose 2-mg regimen. This shortened loading phase potentially reduces the initial treatment burden. Further prospective studies should validate these preliminary outcomes.

### Declaration of generative AI and AI-assisted technologies in the manuscript preparation process

During the preparation of this work the authors used Gemini 3.1 Pro (Google LLC, Mountain View, California, USA) in order to improve the English readability and language of this manuscript. After using this service, the authors reviewed and edited the content as needed and take full responsibility for the content of the published article.

## Supporting information

S1 Table Comparison of baseline covariates between unmatched cohorts. (DOCX)
