# Supplementary material for: Two-dose aflibercept 8 mg versus three-dose aflibercept 2 mg loading for treatment-naïve neovascular age-related macular degeneration
Source: PLoS One. 2026 Sep 11;21(9):e0349947. doi: 10.1371/journal.pone.0349947 (PMC13567766; doi:10.1371/journal.pone.0349947)
Supplement: Supplementary file 1 [file pone.0349947.s001.docx]

| **S1 Table. Comparison of baseline covariates between unmatched cohorts** | | | | |
| --- | --- | --- | --- | --- |
|  | **8 mg** | **2 mg** | ***P* value** | **SMD** |
| Eyes, n | 31 | 82 |  |  |
| Age, years | 78.8 ± 7.6 | 75.0 ± 8.3 | 0.03 | 0.479 |
| Sex, men/women, n | 18/13 | 52/30 | 0.60^†^ | 0.113 |
| Subtype, typical nAMD/PCV/RAP, n | 14/14/3 | 43/32/7 | 0.79^†^ | 0.151/0.121/0.040 |
| LogMAR BCVA | 0.43 ± 0.40 (Snellen, 20/54) | 0.43 ± 0.39 (Snellen, 20/54) | 0.99 | 0.023 |
| Data are presented as means ± standard deviations where applicable.  nAMD = neovascular age-related macular degeneration; PCV = polypoidal choroidal vasculopathy; RAP = retinal angiomatous proliferation; LogMAR BCVA = logarithm of the minimum angle of resolution best-corrected visual acuity.  ^†^, Chi-square test; for the others, the Mann–Whitney U test. | | | | |
